# Insight into the Investigation of Diamond Nanoparticles Suspended Therminol^®^55 Nanofluids on Concentrated Photovoltaic/Thermal Solar Collector

**DOI:** 10.3390/nano12172975

**Published:** 2022-08-28

**Authors:** Likhan Das, Fazlay Rubbi, Khairul Habib, Navid Aslfattahi, Saidur Rahman, Syed Mohd Yahya, Kumaran Kadirgama

**Affiliations:** 1 Department of Mechanical Engineering, Universiti Teknologi PETRONAS, Seri Iskandar 32610, Malaysia; 2 Department of Industrial and Manufacturing Systems Engineering, Iowa State University, Ames, IA 50011, USA; 3 Department of Fluid Mechanics and Thermodynamics, Faculty of Mechanical Engineering, Czech Technical University in Prague, Technická 4, 166 07 Prague, Czech Republic; 4 Research Centre for Nanomaterials and Energy Technology (RCNMET), School of Engineering and Technology, Sunway University, Petaling Jaya 47500, Malaysia; 5 Sustainable Energy and Acoustics Research Lab, Mechanical Engineering Department, Aligarh Muslim University, Aligarh 202002, India; 6 Faculty of Mechanical and Automotive Engineering Technology, Universiti Malaysia Pahang, Pekan 26600, Malaysia; 7 Advanced Nano Coolant-Lubricant (ANCL) Lab, Automotive Engineering Centre, Universiti Malaysia Pahang, Pekan 26600, Malaysia

**Keywords:** nanofluid, thermal properties, optical properties, stability and CPV/T solar collector

## Abstract

Nanofluids are identified as advanced working fluids in the solar energy conversion field with superior heat transfer characteristics. This research work introduces carbon-based diamond nanomaterial and Therminol^®^55 oil-based nanofluids for implementation in a concentrated photovoltaic/thermal (CPV/T) solar collector. This study focuses on the experimental formulation, characterization of properties, and performance evaluation of the nanofluid-based CPV/T system. Thermo-physical (thermal conductivity, viscosity, and rheology), optical (UV-vis and FT-IR), and stability (Zeta potential) properties of the formulated nanofluids are characterized at 0.001–0.1 wt.% concentrations of dispersed particles using experimental assessment. The maximum photo-thermal energy conversion efficiency of the base fluid is improved by 120.80% at 0.1 wt.%. The thermal conductivity of pure oil is increased by adding the nanomaterial. The highest enhancement of 73.39% is observed for the TH-55/DP nanofluid. Furthermore, dynamic viscosity decreased dramatically across the temperature range studied (20–100 °C), and the nanofluid exhibited dominant Newtonian flow behavior, with viscosity remaining nearly constant up to a shear rate of 100 s^−1^. Numerical simulations of the nanofluid-operated CPV/T collector have disclosed substantial improvements. At a concentrated solar irradiance of 5000 W/m^2^ and an optimal flow rate of 3 L/min, the highest thermal and electrical energy conversion efficiency enhancements are found to be 11 and 1.8%, respectively.

## 1. Introduction

Renewable energy has been established as a sustainable resource that can produce clean energy to tackle the dearth of conventional non-renewable energy resources such as carbon-based fossil fuels. Solar energy is one of the renewable resources that is environmentally suitable, non-toxic, viable, and easily accessible due to its abundance in nature [1,2,3,4]. Concentrated photovoltaic/thermal (CPV/T) energy conversion systems are considered a potential solar energy conversion system for renewable energy that can concurrently generate thermal and electrical energy without affecting the environment. A CPV/T solar collector system is a type of concentrated solar system that combines a concentrator, a photovoltaic unit, and a thermal collector to harvest solar energy. Furthermore, because of the concentrated optical design, the CPV/T solar system requires fewer PV cell surface areas and has reduced construction and maintenance costs. The hybrid CPV/T device is a low-emission technology that is commonly used in industrial and residential applications [5]. It is an advanced variant of a traditional CPV collector that does not include a cooling unit and results in overheating issues at higher irradiation intensities, which decreases the device’s performance [6]. 

In CPV/T, the collector’s effective concentrating device concentrates incoming solar irradiation onto the photovoltaic panel’s surface. The thermal collector integrated into the CPV/T acts as a cooling system which maintains the temperature of the photovoltaic panel so that the concentrated heat flux can be converted into electrical power more efficiently [7]. As concentrated solar radiation strikes the surface of the polycrystalline silicon cells of the PV panel, approximately 15 to 20% of the incident solar energy can be converted into electrical energy by the PV unit, and the remaining irradiance is transferred to the working fluid of the thermal cooling unit [8]. In comparison to traditional CPV, non-concentrating hybrid PV/T, and standalone PV devices, hybrid CPV/T collectors produce more electrical power and thermal energy concurrently [9]. However, the inefficiency of conventional heat transfer fluids (HTFs) in converting solar energy to thermal and electrical power limits the cooling and total efficiency of the PV/T collector, thus reducing the output of CPV/T [10]. Therefore, to augment the energy efficiency of solar collectors, such as PV/T and CPV/T, traditional HTF should be replaced with a working fluid that possesses efficient thermal energy conversion properties.

In this context, nanofluids (NFs) with advanced thermo-optical properties have emerged as an alternative technology to improve the energy efficiency of traditional working fluids. NFs/nano-colloids are highly engineered dispersions of solid nanomaterials with a nanoscale (<100 nm) in a liquid base fluid. Due to the advanced properties of dispersed nanoparticles, stable NFs exhibit exceptional photo-thermal properties in comparison to base fluids (NPs) [11,12,13,14]. Due to their superior properties, nanomaterials dispersed in colloidal suspensions provide a remarkable improvement in the thermal and optical properties of NFs. Different types of metals, metal-oxides, carbon-based nanotubes (CNT), and two-dimensional (2D) graphene NPs are extensively utilized for NF formulation [15,16,17]. Among the wide range of NPs, carbon-based materials exhibit greater effectiveness because of their larger surface area and chemical structure relative to non-carbon-based particles [18,19]. Yu, et al. [20] investigated the thermal conductivity of stable graphene—EG nanofluids in comparison to pure EG. Using a two-step technique, the researchers dispersed 2.0 and 5.0 vol.% graphene of 0.7–1.3 nm size in EG to synthesize their nanofluids at temperatures ranging from 10 to 60 °C. At 60 °C. The as-prepared 5.0 vol.% suspension demonstrated an 86% increase in thermal conductivity over the basic fluid. Yarmand, et al. [21] used a two-step technique at 20–40 °C to formulate water-based nanofluids containing 0.02 to 0.1 wt% functionalized graphene nanoplatelets. The functionalization method commenced with an acidic treatment of the graphene powder, which was accomplished by dispersing the graphene in a 1:3 mixture of HNO_3_ and H_2_SO_4_. During their 245-h test, they noticed that the formation of sedimentation within their as-fabricated nanofluids was limited. When using the 0.1 wt% nanofluid, the heat transfer coefficient improved by 19.68% when compared to the base fluid. Furthermore, they determined that the temperature of production and the dispersed solid concentration influence the thermal behavior of the suspension. Apart from heat transport capability, the optical properties, for instance, extinction coefficient and solar absorbance, can be tuned by adding nanoparticles into the conventional heat transfer fluid. Chen, et al. [22] found that adding nanoparticles to an ionic liquid significantly increased the extinction coefficient. When graphene/ionic liquid and SiC/ionic liquid nanofluids are compared, the graphene nanoparticle is found to be more optimistic in improving the extinction coefficient of ionic liquid over the entire wavelength range. Another study by Menbari, et al. [23] reported that increasing the volume fractions of nanoparticles increased the extinction coefficient and absorbency spectra of nanofluids, which were maximal at the nanofluid threshold stability condition. Due to the enhanced thermal, optical, and chemical properties of NFs derived from carbon nanomaterials, such fluids are being applied as cooling fluids in hybrid PV/T energy conversion systems [24,25]. Many experimental and numerical studies on solar energy conversion technology have drawn attention to the potency of NFs for performance enhancement of various concentrating and non-concentrating solar collector systems by utilizing them as working fluids [26,27,28]. 

In an experimental investigation, Qu, et al. [29] examined the photo-thermal energy transformation characteristics of aqueous/CuO + MWCNT NFs for solar energy harvesting applications. The NFs exhibited significant absorption properties and attained a 14.1 °C higher temperature compared to the base fluid. Furthermore, NFs showed advanced optical behavior in response to incoming solar radiation at low particle loading. Recent experimental and numerical studies on solar energy conversion technology have drawn attention to the potency of NFs in enhancing performance in a variety of concentrating and non-concentrating solar systems by utilizing them as working fluids [26,27]. Nonetheless, only a few studies on the implementation of NF-based hybrid PV/T or CPV/T devices at concentrated irradiance have been conducted. Hemmat Esfe, et al. [30] evaluated the effectiveness of NF-based collectors by analyzing a wide range of literature that used pure fluids and NF as the working fluid in hybrid PV/T collectors. They discovered that NF-based systems outperform conventional fluids in terms of electrical efficiency ($\eta_{el}$), PV panel cooling, and thermal efficiency ($\eta_{th}$). Some recent studies on nanofluid-based PV/T and/or CPV/T systems are reviewed in Table 1.

In the present study, a novel combination of Therminol^®^55 and oil diamond particles (DP) at the nanoscale range was used to experimentally formulate and characterize the TH-55/DP NFs for potential application in the CPV/T system. Prepared NF samples are characterized to evaluate their thermo-physical, optical, and chemical properties. Hence, thermal conductivity, specific heat capacity, viscosity, UV-Vis, TGA, FT-IR, and stability analysis are carried out to assess the potency of the NF for parabolic trough solar collector systems. Afterward, the thermal performance of the formulated nanofluid-based hybrid CPV/T system is conducted numerically, considering different important operating conditions (heat flux, flow rate, and particle concentration). This is the first study to examine the experimental formulation, characterization, and numerical application of carbon-based TH-55/DP NF on a CPV/T system to date. Numerical analysis is performed using COMSOL Multiphysics^®^ software (version: 5.6) to evaluate the performance of the system. Examining the NFs, thermal conductivity is found to be augmented with the addition of nanomaterials and elevated temperatures as well. The dynamic viscosity of the NFs decreases rapidly at rising temperatures, which makes the nanofluid particularly suitable for applications in concentrated solar collectors like CPV/T. In the application part of this work, the performance (thermal, electrical, and cooling) of the nanofluid-based hybrid CPV/T system is reported as new findings and compared with the base fluid operating system. Overall, the findings of this study will shed light on the use of TH55-based nanofluid as a viable heat transfer fluid for concentrated solar collectors and other medium-to-high temperature range solar thermal applications.

## 2. Methodology

### 2.1. Materials

Therminol^®^ 55 (TH-55) was purchased from EASTMAN (Kingsport, TN, USA) and used as a pure base fluid to formulate the nanofluid, medium temperature range industry-standard synthetic oil. Carbon-based diamond nanoparticles (purchased from US Research Nanomaterials, Inc., Houston, TX, USA) are utilized to prepare the nanoscale suspension. The particles range in size from 3 to 10 nm and have a purity of 98.3%. The nanoparticles are gray in color and have a spherical shape. The properties of the base fluid and nanoparticles are listed in Table 2.

### 2.2. Formulation of Nanofluid

**Figure 1:**
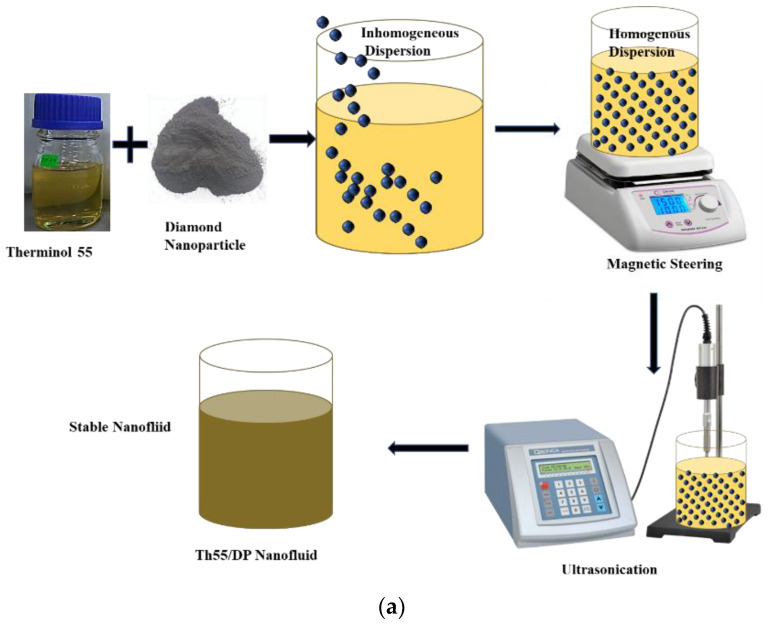

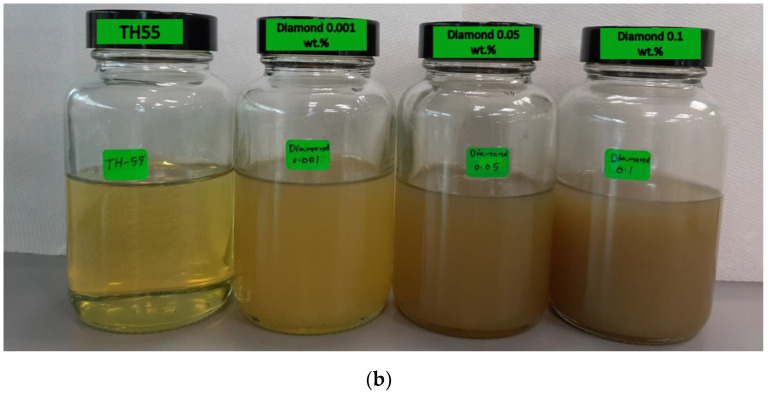
(**a**) Schematic of Two-step Nanofluid Formulation method, (**b**) Formulated TH-55/DP stable nanofluid at different concentrations.

TH-55/DP NF samples are prepared experimentally in two steps at three different particle loadings (0.001, 0.05, and 0.1 wt.%) of diamond nanomaterial. The step-by-step procedures for formulating nanofluid are illustrated in Figure 1a. The two-step method is suitable in terms of commercial aspects and industrial large-scale production of NF. In the first step, the estimated amount of diamond nanoparticles is dispersed into pure TH-55 as per weight fractions. To make the suspension homogenous, several mechanical stabilization techniques are employed in the second step of the formulation process. Immediately after the addition of solid particles into the oil, all the samples are stirred with a magnetic stirrer for about 30 min at 700 rpm and 80 °C. For further stabilization, NFs are sonicated at high frequency (1200 W, 20 kHz) utilizing an ultrasonic homogenizer (FS-1200N) for 30 min. To ensure effective stabilization, the sonication is carried out at a temperature of around 80 °C. This technique provides uniform dispersion of diamond nanoparticles in the TH-55 by distributing the particles evenly throughout the suspension, as depicted in Figure 1b.

### 2.3. Characterization

#### 2.3.1. Structural and Optical Properties Measurement

Nanofluid samples are characterized by employing several instruments for optical, chemical, and morphological investigation. Fourier transform infrared spectroscopy (FT-IR) is conducted to identify the existing functional groups in the suspensions and observe the chemical composition in the suspension of Therminol^®^55 with diamond nanoparticles. The experiment was performed using a high-performance Spectrum-Two^TM^ FT-IR spectrometer from Perkin Elmer (Waltham, MA, USA). The instrument can measure at the highest resolution of 0.5 cm^−1^ for a spectral range of 350 to 8300 cm^−1^ using a single channel LiTaO_3_ detector. Transmittance and absorbance characteristics of TH-55/DP NFs are examined utilizing the UV-Vis Spectrometer for the wavenumber range of 450 to 4000 cm^−1^ at 0.2 cm/s scanning speed. Lambda 750 (Parkin Elmer, Waltham, MA, USA) is employed to accomplish the absorbance characteristic of the formulated NFs using a monochromatic light source of 860 nm. All samples are measured at room temperature for 200 to 800 nm wavelengths at a scanning speed of 266.75 nm/min. Suspension stability of the NFs is examined by using an electrophoresis technique to measure the electrophoretic mobility of dispersed particles in terms of Zeta potential. Anton Paar’s LITESIZER 500 (Graz, Austria) is operated to conduct the assessment using dynamic, electrophoretic, and static light scattering techniques. It offers good measurement accuracy for samples with a size range of 3.8 nm to 100 µm. All the equipment is calibrated before assessing the NF samples, and protocols are repeated several times to ensure consistent precision of the measurements.

#### 2.3.2. Thermo-Physical Properties Measurement

**Figure 2:**
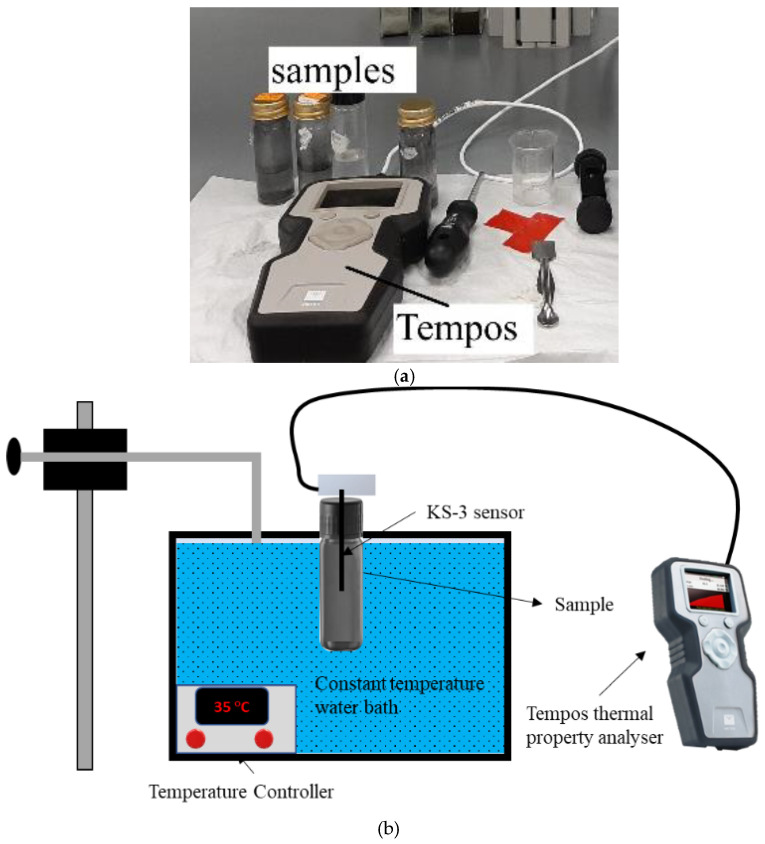
(**a**)Tempos thermal conductivity measuring instrument based on transient hot wire method, (**b**) Schematic of the thermal conductivity measurement setup.

To obtain the thermal conductivity (TC) of TH-55/DP NFs, TEMPOS (from Meter Group USA) is operated at varying temperatures ranging between 30 and 70 °C temperature. Figure 2a depicts the TEMPOS thermal conductivity measuring instrument that was used to measure the TC of the samples in this present experiment. This equipment functions under the transient hot wire method and is supplied with several sensors and a digital controller with a DB-15 connector. In the present experiment with the NFs, the KS-3 sensor (1.3 mm diameter × 60 mm length) is utilized as it is particularly suitable to assess TC in the range of 0.02 to 2 W/m·K. The schematic of the measurement environment is presented in Figure 2b. TEMPOS can keep the heat constant from the source and offers excellent accuracy in the measurements with an uncertainty of ≤±10%. A temperature-stabilized water-bath (MEMMERT, WNB22, Schwabach, Germany) is integrated during the experiment, and the temperature can be controlled using a knob to measure conductivities at each temperature. Before measuring the conductivities of the NF samples, the equipment is calibrated with glycerin samples provided by the suppliers. In this approach, a certain temperature is set for the water-bath and the samples are placed into the water-bath using jigs. The KS-3 needle sensor is placed inside the sample vertically and connected to the analyzer via a cable. The system takes some time to reach the equilibrium state and then conductivity data along with corresponding temperature and thermal resistivity are taken from the TEMPOS digital screen five times at each temperature to ensure precision of the acquired data.

Due to measurement facility limitations, the *c*_p_ of the TH-55/DP NFs is determined using the correlations widely used in reported studies [39] as follows:

(1) $${\left(\rho c_{p}\right)}_{nf}=\varphi {\left(\rho c_{p}\right)}_{np}+\left(1-\varphi \right){\left(\rho c_{p}\right)}_{bf}$$

where, ${\left(c_{p}\right)}_{np}$, ${\left(c_{p}\right)}_{bf}$ and ${\left(c_{p}\right)}_{np}$ are the specific heat capacity (in J/kg. K) of nanomaterials, base fluid and formulated nanofluid, respectively.

Since concentration of dispersed diamond nanoparticles is very low, the density of the TH-55/DP is presumed to be constant and equal to the density of pure Therminol^®^55. Indeed, the effect of nanoparticle loading on the nanofluid’s density can be significant at high loading values. Without considering the effect of density-shift due to the nanoparticle loadings, the specific heat of the TH-55/DP nanofluid is measured as follows:

(2) $${\left(c_{p}\right)}_{nf}≅{\left(c_{p}\right)}_{np}+\left(1-\varphi \right){\left(c_{p}\right)}_{bf}$$

The viscosity characteristics of pure TH-55 oil, and the solo and hybrid NFs are studied using the MCR-92 Rheometer from Anton Paar (Graz, Austria). The measurements are conducted with respect to a temperature scale of 20 to 100 °C and a shear rate of 30 to 100 s^−1^. The instrument offers very good accuracy (precision: ±1%) for a wide range of viscosity from −40 to 400 °C. The device is calibrated with water before performing the test with oil samples. About 60 mL of each sample is required to measure viscosity with MCR-92. The viscosity was measured at least three times at each temperature to check the measurement repeatability and the outcome was satisfactory with less than 3% deviation.

### 2.4. Numerical Implementation on Concentrated PV/T Solar Collector

In a concentrated photovoltaic/thermal (CPV/T) system, Fresnel lenses are used to focus the sunlight on the poly-crystalline-silicon solar cells of the PV module. Researchers used a variety of active and passive techniques to reduce the temperature rise of photovoltaic module cells. Some of them used nanofluid as a coolant at the back of the photovoltaic panel to reduce the heat and thus boost electrical and thermal efficiency. This section conducts a numerical analysis of the newly produced TH-55/DP based nanofluid in the CPV/T collector. 

**Figure 3:**
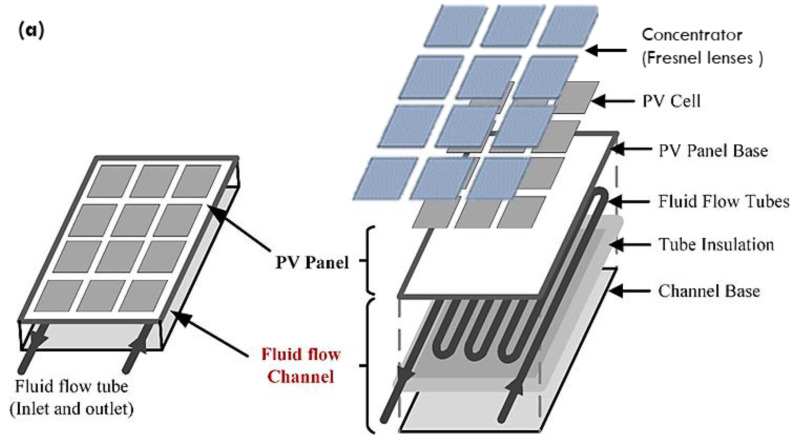

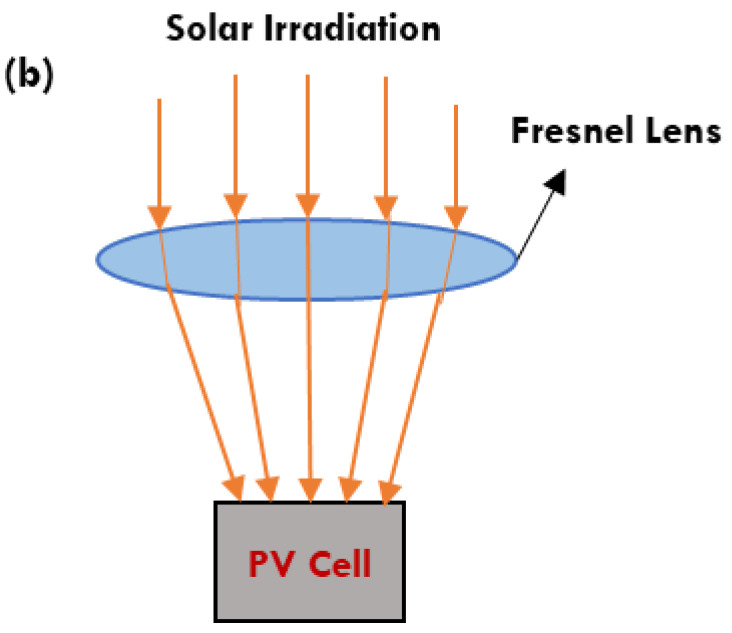
Schematic illustration of (**a**) the intended nanofluid operated PV/T system, (**b**) concentrated solar irradiations on the surface of each PV cell.

The problem under investigation is presented in Figure 3a,b. This investigation considers a large photovoltaic module with 72 polycrystalline silicon cells (each cell has an area of 0.024 m^2^). In India, the average solar radiation is 1000 W/m^2^. To achieve solar irradiation of up to 5000 W/m^2^, each lens must have a surface area of 5 m × 0.024 m, taking the total surface area of the 72 lenses to 72 × 0.122 m^2^. The function of a converging Fresnel lens to concentrate the solar irradiation on the surface of each PV cell is depicted in Figure 3b. The computational domain for numerical simulation is the total area of the solar cells (1.73 m^2^). Table 3 summarizes the physical properties of the PV/T module’s various layers.

#### 2.4.1. Numerical Modelling of CPV/T Solar System

The solar collector under investigation is a 300-watt photovoltaic module made up of four layers: a photovoltaic solar cell, EVA on both sides of the photovoltaic panel, and a tedlar plate. A serpentine copper tubing heat exchanger is placed underneath the photovoltaic module (Figure 1). The PV cells, EVA, and tedlar layers are each 0.3 mm, 0.5 mm, and 0.1 mm thick. The remaining specifications are identical to those of the photovoltaic plate, i.e., (1955 mm × 982 mm). The numerical analysis is carried out using the Finite Element Method-based Multiphysics Software COMSOL. COMSOL’s CFD and heat transfer modules are used to determine the CPV/T system’s output parameters. The nanofluid flow is presumed to be constant, 3D, incompressible, and laminar. The transmissivity of EVA is assumed to be approximately 100%, dust’s effect on the absorptivity of the PV surface is assumed to be negligible, and temperature variation along the module’s thickness is assumed to be zero. Additionally, it is presumed that the TH-55 contains a homogeneous mixture of nanoparticles (i.e., no particle sedimentation). In this study, TH-55/DP based nanofluid with varying nanoparticle concentrations was used. The thermal conductivity, which is proportional to the weight fraction at various temperatures, is converted to a third-order polynomial equation using regression data analysis and then incorporated into the COMSOL through a user-defined function (UDF).

Regression analysis of experimental data is used to model temperature dependence on the viscosity of TH-55/DP nanofluids. Like thermal conductivity, the viscosity correlations are integrated into COMSOL’s CFD environment through the UDF and used for simulation purposes. Heat conduction equations are used to account for heat transfer in the solid domain of the PV/T device. Heat transmission from the surface of the photovoltaic panel to the flow channel is established using the heat conduction equation shown below in Equations (3)–(5) [40].

(3) $$-\left(\frac{k}{\rho C_{p}}\right)\left(\frac{\partial^{2}T}{\partial x^{2}}+\frac{\partial^{2}T}{\partial y^{2}}+\frac{\partial^{2}T}{\partial z^{2}}\right)=\alpha_{p}G-E_{e}-h_{panel-ted}\left(T_{penal}-T_{ted}\right)$$

It reflects the heat transfer between the photovoltaic panel and the tedlar plate. Similarly, additional thermal energy equations for other layers can be presented. Here, $\alpha_{p}$ represents the panel’s absorptivity, *G* is the effective irradiance, $E_{e}$ is the output electrical power and $h_{panel-ted}$ is the coefficient of heat transfer between the PV module and tedlar plate. Correspondingly, other coefficients between the layers are specified in Equations (3)–(5). Specifications of the PV/T collector are listed in Table 3.

From tedlar to serpentine tubing:

(4) $$-\left(\frac{k}{\rho C_{p}}\right)\left(\frac{\partial^{2}T}{\partial x^{2}}+\frac{\partial^{2}T}{\partial y^{2}}+\frac{\partial^{2}T}{\partial z^{2}}\right)=-h_{penal-ted}\left(T_{p}-T_{td}\right)-h_{ted-tubing}\left(T_{ted}-T_{tubing}\right)$$

From serpentine tubing to nanofluid:

(5) $$-\left(\frac{k}{\rho C_{p}}\right)\left(\frac{\partial^{2}T}{\partial x^{2}}+\frac{\partial^{2}T}{\partial y^{2}}+\frac{\partial^{2}T}{\partial z^{2}}\right)=-h_{ted-tubing}\left(T_{ted}-T_{tubing}\right)-h_{tubing-nf}\left(T_{tubing}-T_{nf}\right)$$

For flow in the collector, the coupled heat transfer equation is used, as both conduction and convection are considered in Equation (6). Moreover, Equations (7)–(10) describe the mass and momentum equations for laminar fluid flow.

(6) $$\rho_{nf}C_{Pnf}\left(u\frac{\partial T}{\partial x}+v\frac{\partial T}{\partial y}+w\frac{\partial T}{\partial z}\right)=K_{nf}\left(\frac{\partial^{2}T}{\partial x^{2}}+\frac{\partial^{2}T}{\partial y^{2}}+\frac{\partial^{2}T}{\partial z^{2}}\right)$$

(7) $$\frac{\partial u}{\partial x}+\frac{\partial v}{\partial y}+\frac{\partial w}{\partial z}=0$$

X-momentum:

(8) $$\rho_{nf}\left(u\frac{\partial u}{\partial x}+v\frac{\partial u}{\partial y}+w\frac{\partial u}{\partial z}\right)=\frac{-\partial P}{\partial x}+{µ}_{nf}\left(\frac{\partial^{2}u}{\partial x^{2}}+\frac{\partial^{2}u}{\partial y^{2}}+\frac{\partial^{2}u}{\partial z^{2}}\right)$$

Y-momentum:

(9) $$\rho_{nf}\left(u\frac{\partial v}{\partial x}+v\frac{\partial v}{\partial y}+w\frac{\partial v}{\partial z}\right)=\frac{-\partial P}{\partial y}+{µ}_{nf}\left(\frac{\partial^{2}v}{\partial x^{2}}+\frac{\partial^{2}v}{\partial y^{2}}+\frac{\partial^{2}v}{\partial z^{2}}\right)$$

Z-momentum:

(10) $$\rho_{nf}\left(u\frac{\partial w}{\partial x}+v\frac{\partial w}{\partial y}+w\frac{\partial w}{\partial z}\right)=\frac{-\partial P}{\partial z}+{µ}_{nf}\left(\frac{\partial^{2}w}{\partial x^{2}}+\frac{\partial^{2}w}{\partial y^{2}}+\frac{\partial^{2}w}{\partial z^{2}}\right)$$

The density ($\rho_{nf}$) and heat capacity ($C_{Pnf}$) of the nanofluid are considered to be constant, and their properties were obtained from experiential correlations [42]:

(11) $$\rho_{nf}=\left(1-\phi \right)\rho_{bf}+\phi \rho_{s}$$

(12) $$Cp_{nf}=\left(1-\phi \right){\left(C_{P}\right)}_{bf}+\phi {\left(C_{P}\right)}_{s}$$

Energy conservation is considered throughout the hybrid PV/T collector described in Equation (13), which includes solar irradiance, PV surface emission, convection between the PV/T and the surrounding atmosphere, thermal energy produced, and electrical power production.

(13) $$G-P_{el}-P_{th}-Q_{conv}^{\prime }-Q_{rad}^{\prime }=0$$

The following equations describe the convection and radiation heat transport from a PV/T device. The convective and radiative heat transfer coefficients from the panel are determined using Newton’s cooling and the Stefan-Boltzmann laws, respectively.

(14) $$-n\cdot \left(-k\nabla T\right)=h_{total}\left(T_{surface}-T_{ambient}\right)$$

(15) $$-n\cdot \left(-k\nabla T\right)=\varepsilon \sigma \left(T_{surface}^{4}-T_{sky}^{4}\right)$$

where $h_{total}$ denotes the total heat transfer coefficient expressed in terms of $h_{total}={\left(h_{forced}^{3}+h_{natural}^{3}\right)}^{\frac{1}{3}}$. This involves both natural and induced convection effects on the panel. The coefficients of induced and natural convection heat transport [43] are determined using Equations 16 and 17.

(16) $$h_{natural}=1.78{\left(T_{amb}+T_{surface}\right)}^{\frac{1}{3}}$$

(17) $$h_{forced}=2.8+3.0V_{wind}$$

While sky temperature is determined using the Swinbank relation [44] as $T_{sky}=0.037536T_{amb}^{4}+0.32T_{amb}$. In Equation (15), ε is the emissivity and σ denotes as constant of Stefan-Boltzmann.

The gained electrical and thermal output are calculated by:

(18) $$P_{el}=V_{oc}\times I_{sc}\times FF$$

(19) $$P_{th}=mC_{p}\left(T_{out}-T_{in}\right)$$

The fill factor (FF) value was 0.8 for the considered solar cell.

Equations (20) and (21) determine the electrical and thermal efficiency, respectively.

(20) $$\eta_{el}=\frac{P_{el}}{G\times A_{c}}$$

(21) $$\eta_{th}=\frac{P_{th}}{G\times A_{c}}$$

#### 2.4.2. Boundary Conditions

Proper boundary conditions were used throughout the domain in accordance with the physics of the problem. The boundary condition that is applied across the top and bottom layers of the photovoltaic module is $-n\cdot q=h_{c}\left(T_{amb}-T_{s}\right)$. Where *n* is the surface normal and $T_{amb}$ and $T_{s}$ are the surrounding environment and surface temperatures, correspondingly. For the fluid domain, the inlet boundary condition is specified as the velocity of the inlet along the x-axis i.e., *u* = *U_0_*, *v* = 0, *w* = 0 and *T* = *T_0_*. For solid boundaries, no-slip condition is used (*u* = *v* = *w* = 0). However, at the outlet, a zero-pressure boundary condition is used (*p* = 0). For solid-fluid interface heat flux continuity at the interface is used ${\left(\frac{\partial T_{s}}{\partial n}\right)}_{f}=\frac{k_{s}}{k_{f}}{\left(\frac{\partial T_{s}}{\partial n}\right)}_{s}$, adiabatic boundary condition is applied to the solid walls of the device. Furthermore, the lowermost plate of the collector remains isolated.

#### 2.4.3. Meshing and Grid Independency Test

**Figure 4:**
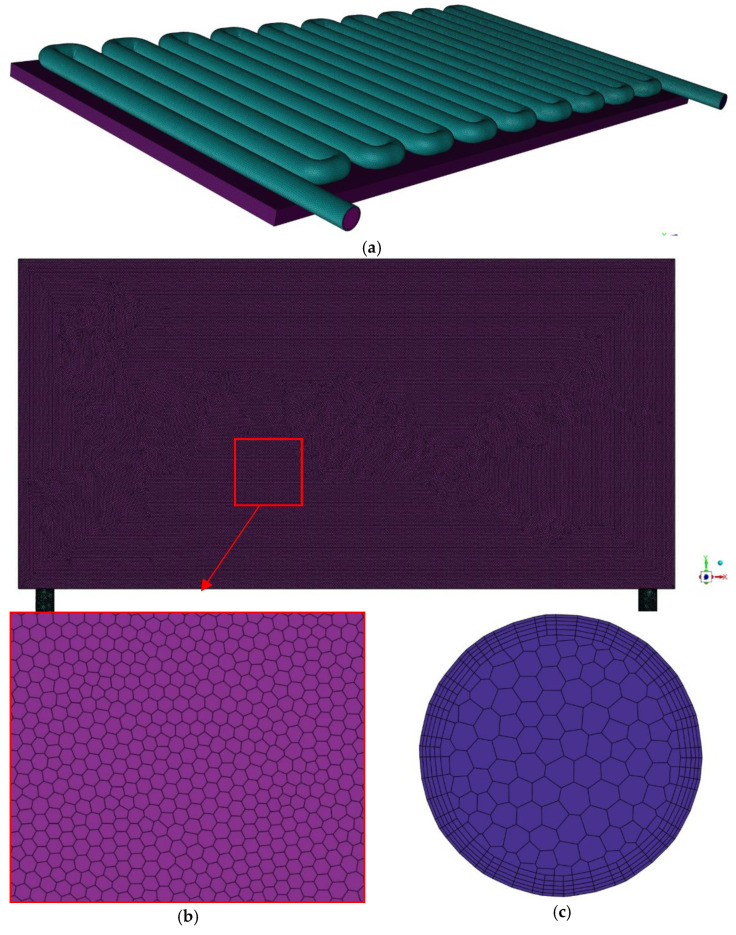
(**a**) 3D grid generation of the computational domain, (**b**) Poly-hexacore meshing of the PVT system, (**c**) Inflation layer at the solid-fluid interface.

The meshing of the PV/T model was accomplished utilizing the ANSYS meshing interface, which was later solved in COMSOL Multiphysics^®^. The Ansys meshing interface is a user-convenient and structured poly-hexacore mesh geometry, which is preferred as the meshing element in this current geometry cannot be obtained using the COMSOL meshing interface (Figure 4). Using poly-hexacore meshing, the grid convergency can be obtained at a much lower element size than using unstructured meshing. Thus, simulation time and the number of iterations are substantially reduced to obtain more accurate results. In order to capture the boundary layer effect, the solid liquid interfaces are treated with an inflation layer with a growth rate of 1.2. At each boundary, the number of mesh elements increases to provide for accurate resolution of the heat transfer and flow fields. For grid independence, simulations are run with water as the coolant at 1000 W/m^2^ and a flow rate of 3 LPM for various mesh sizes, from coarse to fine, as indicated in Table 4. The primary layer depth is set to 1/50 of the element’s size at the boundary. After mesh no.5, the PV temperature and outlet fluid temperature data were nearly constant. As a result, mesh no.5 was used for simulation purposes.

## 3. Results and Discussion

### 3.1. Stability of the Nanofluids

The homogeneous suspension or the nanofluid’s long-term stability is a critical precondition for industrial applications. In general, the instability of nanofluid is caused by several attraction and repulsion forces acting at the solid-fluid interface of the suspension. When attraction forces dominate, the nanoparticles tend to form clusters and degrade the homogeneity and thermo-optical properties of the suspension. Zeta potential (ζ) analysis is an effective way to determine the dispersion stability of nanomaterials in a fluid medium. In this method, the electrical potential difference (i.e., Zeta potential, ζ) is measured in mV at the electric double layer (EDL) formed at the solid-fluid interface of the suspension [45]. Higher values of ζ imply higher mobility, i.e., the dispersion stability of the particles in the fluid medium. The ζ value beyond ±60 mV represents excellent stability of suspension, whereas values below ±15 mV indicate unstable or poor dispersion of nanoparticles in the mixture [46]. The suspension stability of the prepared NFs is characterized by varying particle wt.% (0.001–0.1) and temperature (25 and 80 °C). The acquired ζ results (depicted in Table 5) imply good stability of the formulated fluids, particularly at the lowest concentration (0.001 wt.%). The addition of further particles (0.05 and 0.1 wt.%) reduced the suspension homogeneousness as excess particles led to the formation of clusters in the fluid medium. On the third day of preparation, absolute values of ζ at 25 °C were measured as 45.13, 41.21, and 34.81 mV for TH-55/DP samples at 0.001, 0.05, and 0.1 wt.%, respectively.

In terms of temperature, it has been observed that NFs are more stable at higher temperatures than at lower temperatures. At 80 ℃, NFs exhibited slightly higher absolute ζ relative to 25 ℃ for all three concentrations. The results are consistent with reported studies with similar NFs [34,47].

### 3.2. Optical Properties

**Figure 5:**
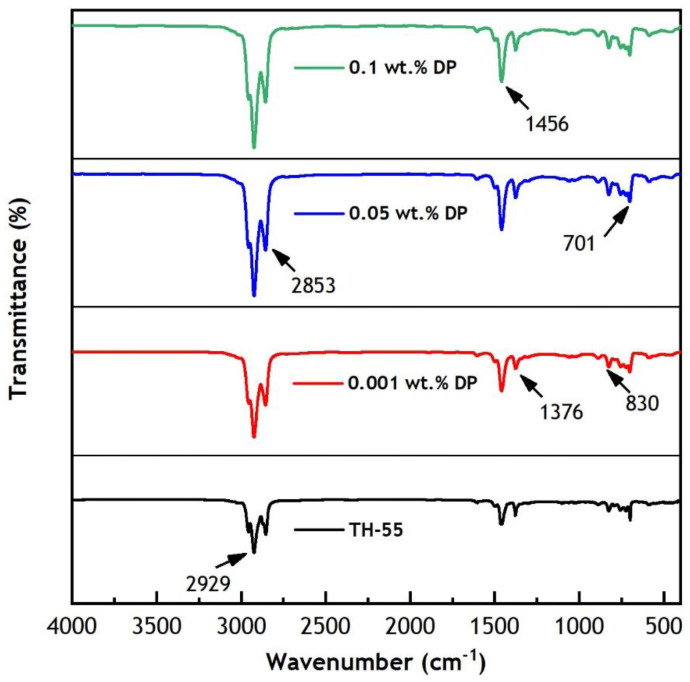
FT-IR spectra of TH-55 and TH-55/DP nanofluids.

Figure 5 represents FT-IR spectra of TH-55 and TH-55/DP NFs at 0.001–0.1 wt.% particle loadings of DPs to assess existing chemical bonds in the fluids. The study is performed for each sample from 450 to 4000 cm^−1^ wavenumber at room temperature. The NFs depicted similar profiles of the spectrums with no major variation in the absorption peaks as particles are suspended in the identical base fluid. This suggests no chemical reaction between the fluids and particles except physical interactions. Therefore, the present optical results of TH-55/DP NFs confirm that the fluids are consistent in terms of chemical stability.

Several absorption peaks are noticed from the mixture of TH-55 oil and nanomaterial in the spectra absorbing IR bands. Key absorption peaks were observed in the spectrums at wavenumbers of 2929 cm^−1^, 2853 cm^−1^, 1456 cm^−1^, 1376 cm^−1^, 830 cm^−1,^ and 701 cm^−1^. The corresponding peaks at 2929 cm^−1^ and 1456 cm^−1^ revealed the presence of CH_3_ and CH_2_ stretching bonds in the synthetic oil chain of TH-55, respectively [48]. The bands at 1459 cm^−1^ and 1376 cm^−1^ are identified as C=C and C=O stretches of the carbon skeleton due to the dominance of the oil in the mixture [47]. The peaks at a low frequency of 701 cm^−1^ are attributed to C–H bending vibration of the suspension [48]. No discrepancy is observed in terms of the deviation of absorption peaks changes in FT-IR spectrum profiles among the oil and NF suspensions. The conclusive results imply that the formulated NFs are chemically stable.

**Figure 6:**
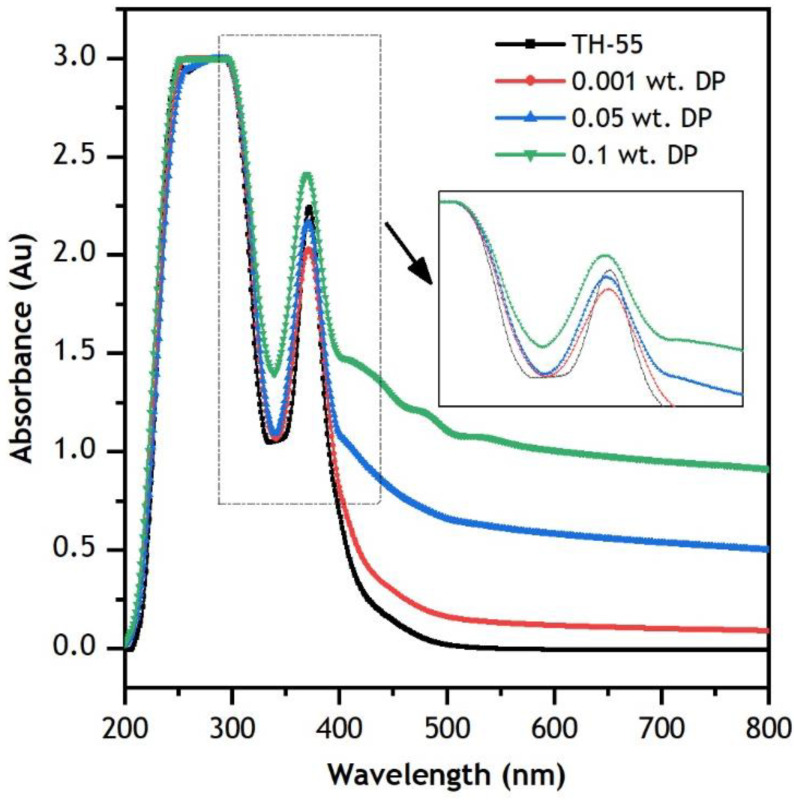
Optical UV−vis absorbance results of TH−55 and TH−55/DP nanofluids.

Diamond nanoparticles are suspended into TH-55 to enhance the absorption aptitude of the fluids so that it can improve the photo-thermal and cooling efficiencies of the hybrid CPV/T system. The optical absorption characteristics of formulated TH-55/DP NFs are examined by analyzing the UV-vis spectrums presented in Figure 6. The analysis was performed with TH-55 and several concentrations (0.001, 0.05, and 0.1 wt.%) of DP for a wavelength range of 200 to 800 nm as it contains over 80% of the total solar radiation emitted from the sun. According to the law of Beer-Lambert, the absorption property of fluid will be augmented due to the addition of solid particles into it and absorbance should be improved with increasing weight loadings of particles dispersed into the fluid [49]. Figure 6 exhibits absorption of TH-55/DP NFs intensified, notably at different peaks and on a visible wavelength scale relative to pure TH-55. Absorbance enhanced rapidly at 200–250 nm and observed high light absorbance up to 450 nm wavelength. At higher wavelengths (450–800 nm), absorbance remained steady for all the fluids, and the absorbance of NF samples remained greater than TH-55 oil. Gulzar, et al. [50] observed an analogous trend in absorption characteristics while investigating several TH-55 based NFs. The obtained average increments in absorbance for TH-55/DP NF at 0.001, 0.05, and 0.1 wt.% are 16.18, 65.28, and 120.80%, respectively. The remarkable intensification in photo-thermal efficiency is due to the absorption capacity and larger surface area of diamond particles that lead to advanced light-to-heat energy conversion behavior by the NFs [48]. The results from the UV-vis analysis indicate the superior absorption capability of the formulated NF and undoubtedly validate its potential implementation on direct absorption solar collectors, for instance, a hybrid CPV/T solar system. The findings are consistent with other nanofluids as well [51,52].

### 3.3. Thermo-Physical Properties

#### 3.3.1. Thermal Conductivity (k)

**Figure 7:**
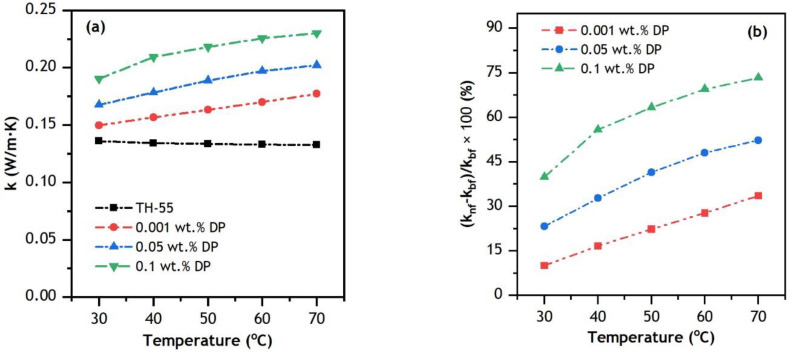
Thermal conductivity (**a**) variation and (**b**) enhancement of TH-55 and TH-55/DP NFs.

Experimental results on k variation of TH-55/DP NFs at 0.001–0.1 wt.% concentrations of nanoparticles against the raised temperature (30 to 70 °C) are elucidated in Figure 7. The TC values of the NFs are measured experimentally within a standard deviation of < ±0.0025. The addition of diamond particles in TH-55 provides a significant augmentation in the thermal conductivity of the fluid. Furthermore, as the temperature was raised, the TC of the NFs improved, whereas the TC of the TH-55 without nanoparticles degraded. Figure 7a,b depicts the percentage linear increment of TC for formulated NFs with the inclusion of nanoparticles as well at increasing temperatures. The enhancement is calculated using the equation $\left(\frac{k_{nf}-k_{bf}}{k_{bf}}\right)\times 100\%$ where, $k_{nf}$ is TC of NF and $k_{bf}$ is TC of TH-55. At 70 °C, the maximum increment in TC of TH-55/DP NFs is 33.60, 52.29 and 73.39% at 0.001, 0.05 and 0.1 wt.%, respectively. The remarkable improvement of TC for all the NFs is due to the large conductive surface area of diamond particles, and stable dispersion and movement of the suspended particles [53]. Added particle wt.% is observed to have a noteworthy impact on TC as higher wt.% yields more conductivity of the NFs. Thus, it is evident that dispersed solid particles offer effective heat transport aptitude in comparison with pure base fluid.

The phenomenon of TC improvement with increasing temperature is associated with particle Brownian motion and kinetic energy, which increases heat transport through dispersed nanomaterials [54,55]. As Brownian motion intensifies at higher temperatures, it enables heat to be transported more efficiently from one particle to another, utilizing the large surface area of the particles. The TC of the NFs intensified more at higher temperatures due to effective Brownian movement at elevated temperatures. Similar findings are reported by other carbon material-based NFs [56,57]. As intensified TC of the working fluid can produce an enhanced thermal performance of the system, the formulated NFs can be potential HTFs to utilize in thermal systems like CPV/T solar collectors.

#### 3.3.2. Dynamic Viscosity

**Figure 8:**
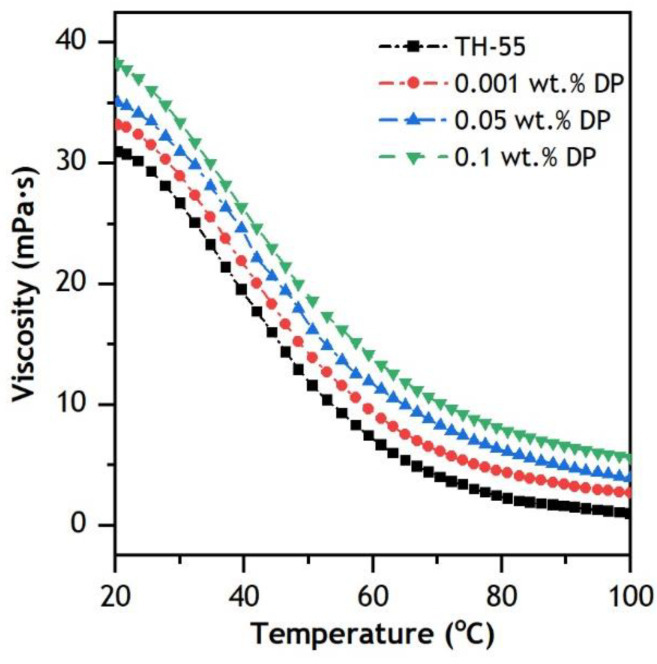
Variation of dynamic viscosity of TH-55/DP nanofluids at 20–100 °C.

Dynamic viscosity (μ) is one of the key properties of NF, which is defined by the resistance force that causes fluid deformation in the reverse path of flow. It has a substantial effect on heat transfer by convection in fluids during the application of fluids to thermal systems. This is mainly due to its impact system’s pumping power. Lower μ of working fluids results in less pumping power being required to operate solar thermal systems [58]. The μ of synthesized NFs is measured at considered concentrations (0.001–0.1 wt.%) over a range of temperature (20–100 °C) and shear rate (0–100 s^−1^). The μ of TH-55 varies with the addition of diamond nanomaterials, increasing with the inclusion of solid particles and decreasing rapidly at rising temperatures (Figure 8). The drop in μ is due to weaker intermolecular interaction (i.e., adhesion forces) between the fluid and solid materials and higher molecular movement at elevated temperatures. For the temperature variation from 20 to 100 °C, μ reduced from 33.02 to 2.69, 35.19 to 3.93, and 38.48 to 5.61 mPa at 0.001, 0.05 and 0.1 wt.%, respectively. The viscosity values of the NFs at elevated temperatures would provide a potential low-pressure drop while operating in the thermal system relative to low temperatures.

**Figure 9:**
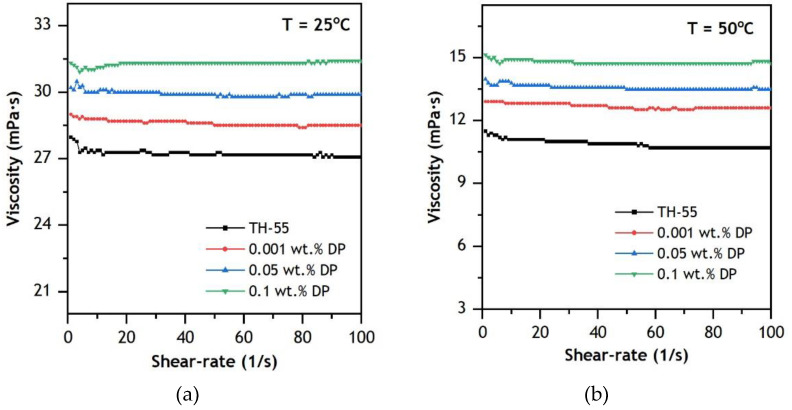
Rheological behavior of TH-55/DP nanofluids at 0–100 s^−1^ shear rate: (**a**) 25 °C and (**b**) 50 °C.

Rheology can be defined as the flow behavior of fluids streaming under induced tensions. To characterize the rheological characteristic of experimentally formulated NFs, variation in μ of the NFs is observed for the corresponding value of the shear rate at a constant temperature of 25 and 50 °C (Figure 9). The results suggest that Newtonian flow characteristic is dominant in the NFs as μ remained constant over the range of shear rate (0–100 s^−1^) except with a little fluctuation at the lower range of shear rates up to 20 s^−1^. Furthermore, the rheological property remained constant regardless of temperature variation. The Newtonian shear behavior of the NFs is a result of spindle rotation and decorated fluid molecules in the TH-55/DP samples. Hence, further increment in shear rate will not alter the μ of the fluids. The obtained results are in accord with other oil-based NFs reported at similar operating conditions [59,60]. Since these NFs exhibit prominently low μ and Newtonian flow characteristics at elevated operating temperatures, the NFs can be an efficient working fluid in the CPV/T solar system.

#### 3.3.3. Numerical Results

To keep the PV panel temperature within the allowable limit, TH-55/DP based NFs were used in this study. The effects of various operational parameters on the cell efficiency under the impact of five effective suns on a densely packed concentrated photovoltaic system were obtained. The numerical simulations were performed at 1000–5000 W/m^2^ intensity levels, with varying nanoparticle concentrations and at a fixed flow rate of 3 LPM. The obtained temperatures of the PV cells at an irradiation intensity of 1000 W/m^2^ at three different flow rates from the current numerical model, were validated and compared with those of [41] and Rahman, et al. [8] (see Table 6). Furthermore, the thermal and electrical efficiency of the present model is compared with that of Nasrin, et al. [58] (Table 7), which expresses a very good accord of the achieved data with the experimental and numerical data.

**Figure 10:**
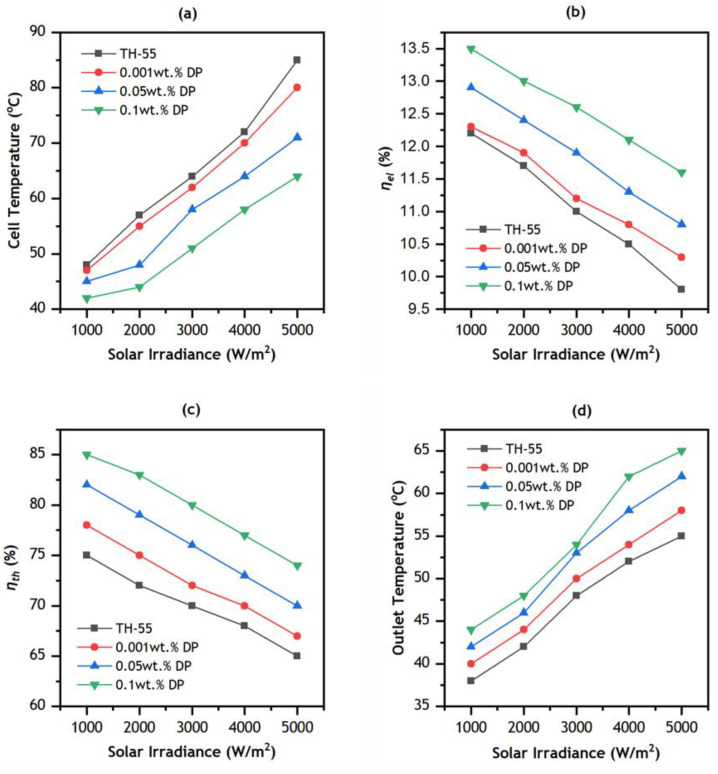
Variation of (**a**) cell temperature, (**b**) electrical efficiency, (**c**) thermal efficiency, and (**d**) outlet temperature, v/s irradiance at 3LPM.

Figure 10a depicts the cell temperature variation with respect to irradiance. The average cell temperature increases with the increase in irradiance intensity from 1000 to 5000 W/m^2^ for each of the nanofluid samples. It was observed from previous studies [40,44] that a rising flow rate beyond 3 LPM results in additional pumping power with little variation in cell temperature. Thus, for the PV/T system, the optimum volume flow rate of cooling NF is 3 LPM. However, further temperature reduction is achieved by NF at higher concentrations of nanoparticles (0.1 wt.%). It was observed that at an irradiance level of 5000 W/m^2^ the solar cell temperature decreases from 85 to 80 °C for TH-55/DP (0.001 wt.%) and from 85 to 71°C for TH-55/DP (0.05 wt.%) and from 85 to 64 °C for TH-55/DP (0.1%). It was observed that for every 100 W/m^2^ rise in irradiance level, cell temperature increases by 0.74 °C for TH-55 cooling fluid. This rise in the case with TH-55/DP (0.1 wt.%) is 0.44 °C.

Figure 10b shows the variation in electrical efficiency ($\eta_{el})$ with irradiance level. Here, the flow rate is fixed at 3 LPM, and irradiance is varied up to 5000 W/m^2^. The $\eta_{el}$ decreases with rising irradiation intensity. It depreciates from 12.2 to 9.8% due to increasing solar radiation for TH-55, from 12.3 to 10.3% for TH-55/DP (0.001 wt.%) from 12.9 to 10.8% for TH-55/DP (0.05 wt.%) and from 13.5 to 11.6% for TH-55/DP (0.1 wt.%). Nasrin, et al. [31] showed that the $\eta_{el}$ for Water/Ag nanofluid in PVT system at 5000 W/m^2^ and 3 LPM was 12.5%. This clearly indicates that our nanofluid is performing well.

Figure 10c shows that the percentage of thermal efficiency goes down as the level of irradiation goes from 1000 to 5000 W/m^2^. This is mainly since the total amount of receiving heat from the PV/T system is increased for the purpose of intensifying irradiation intensity. The $\eta_{th}$ of CPV/T system decreases from 78 to 67% for TH-55/DP (0.001 wt.%), from 82 to 70% for TH-55/DP (0.05 wt.%) and from 85 to 74% for TH-55/DP (0.1 wt.%). Nasrin, et al. [44] reported a 0.3% reduction of $\eta_{th}$ for per 100 W/m^2^ increments of intensity. In the case of TH-55/DP (0.1 wt.%), a 0.22% decrement is observed for every 100 W/m^2^ increments in irradiation level. Figure 10d shows the influence of irradiance intensity and diamond nanoparticle loading on the outlet temperature of the nanofluid-operated CPV/T collector. As increased irradiance intensity rises the average temperature of the PV module, that leads to an increase in the amount of heat collected by the cooling NF. In addition, as the nanofluids exhibited superior thermal properties at higher wt.% of the diamond nanoparticles, it resulted in increased cooling for the PV module and increased heat collection by the TH-55/DP. The highest outlet temperature of 65℃ is achieved by TH-55/DP (0.1 wt.%) at an irradiation of 5000 W/m^2^. This effect is also more noticeable at higher irradiance levels as compared to a lower one.

## 4. Conclusions

In this study, TH-55/DP NFs are formulated experimentally from carbon-based diamond nanoparticles at 0.001 to 0.1 wt.%. Important results are obtained in terms of the thermal, optical, and stability behavior of the NFs. Numerical analysis of the implementation of the NFs as the working fluid of the CPV/T system showed significant improvements in cooling, thermal, and electrical efficiency. The investigation was performed considering various nanomaterial loadings (0.001–0.1 wt.%), solar irradiations (1000–5000 W/m^2^), and a flow rate of 3 LPM to evaluate the performance. The major experimental and numerical findings of this work are outlined in the following points:

- Zeta potential (ζ) analysis confirms good suspension stability of the formulated NFs as absolute ζ values are found to be above ±30 mV. At 25 °C, 45.13, 41.21 and 34.81 mV of ζ are measured at 0.001, 0.05 and 0.1 wt.%, respectively. UV-vis profiles of the NFs showed enhanced absorbance of the NFs. The photo-thermal energy conversion efficiency of TH-55 oil has increased by 120.80% due to the dispersion of diamond nanoparticles.
- The addition of nanomaterial to the TH-55 increases thermal conductivity at elevated temperatures ranging from 20 to 70 °C, whereas the addition of nanoparticles increases dynamic viscosity marginally. However, it rapidly drops at elevated temperatures and behaves like a Newtonian fluid.
- Implementing the NFs in the CPV/T solar collector produced an improved cooling effect on the PV unit of the system as a maximum temperature drop of 21 °C was attained at 0.1 wt.% of DP. The highest augmentation in $\eta_{th}$ and $\eta_{el}$ of the nanofluid-based CPV/T system was obtained at about 11% and 1.8% at an optimum flow rate of 3 LPM, respectively.
- Future research could investigate the practical implementation of proposed nanofluids on CPV/T solar collectors by examining additional parameters, such as exergy analysis and estimation of the system’s pumping power.

## Figures and Tables

**Table 1:** Summary of recent studies on PV/T or CPV/T system using different nanofluids at various operating conditions.

Study	Nanofluids	Dimension and Concentration (φ)	Solar Irradiance	Temperature Range	Flow Rate	Important Findings
Nasrin, et al. [31]	Water/Cu, Ag and Al	-- Φ = 0.5–3 vol.%	5000 W/m^2^	20–140 °C	180 L/h	- The nanofluids exhibited optimized perfor-mance at 2 vol.% concentration. The $\eta_{th}$ is augmented by 7.49, 7.08 and 4.97% for PV/T operated by water/Ag, water/Cu, and water/Al NFs, respectively, relative to water.
Bellos and Tzivanidis [32]	Syltherm-800/CuO	-- Φ = 5 vol.%	1000 W/m^2^	25–200 °C	300–720 L/h	- 1.66, 5.17, 3.05 and 2.08% enhancements were obtained in $\eta_{th}$$,\;\eta_{el}$$,\;\eta_{exergy}$ and $\eta_{overall}$, respectively.
Alous, et al. [33]	Water/Graphene and MWCNT	0.55–1.2 nm 18–28 nm respectively. Φ = 0.5 wt.%	Up to 1000 W/m^2^	20–80 °C	0.5 L/min	- The PV surface temperature was reduced by 14 °C with graphene nanoplatelets and 16 °C with MWCNT NFs. In comparison to PVT-water, the NF-based PV/T had an improved average daily total energetic efficiency of 18.0 and 7% for graphene and MWCNT NFs, respectively.
Rubbi, et al. [34]	Soybean oil/ MXene	1–10 µm × 1 nm Φ = 0.025–0.125 wt.%	1000 W/m^2^	25–80 °C	0.01–0.07 kg/s	- $\eta_{th}$ and $\eta_{el}$ augmented by 15.51 and 15.41% relative to alumina NF. Temperature of the PV unit dropped by 14 °C relative to conventional PV/T.
Kazem, et al. [35]	Water-EG/SiC	45–65 nm Φ = 0.1–4 wt.%	200–1000 W/m^2^	25–70 °C	10–40 kg/h	- PV-cell temperature dropped by 11.34%, while $\eta_{th}$ and $\eta_{el}$ improved by 14.3 and 11.7% using the NF as working fluid instead of water.
Aslfattahi, et al. [36]	Silicon oil/ MXene	1–10 µm × 1 nm Φ = 0.05–0.1 wt.%	1000–8000 W/m^2^	30–200 °C	0.005 kg/s	- PV panel temperature lessened by 12.45% and 11.92% enhanced energy output was achieved for the collector using NF at 0.1 wt.%.
Huaxu, et al. [37]	Glycol/ZnO	-- Φ = 11.2–89.2 ppm	Up to 861 W/m^2^	--	--	- Photo-thermal efficiency improved by 47% adding ZnO NPs from 11.2 to 89.2 ppm. 3.8% higher energy conversion efficiency is achieved relative to conventional CPV.
Khanjari, et al. [38]	Water/Al_2_O_3_	-- Φ = 2 vol.%	200–800 W/m^2^	30–70 °C	0.00136 kg/s	- $\eta_{th}$ enhanced by 10% using the NF compared to water operated PV/T. However, $\eta_{el}$ declined at higher absorbed solar radiation.
Nasrin, et al. [31]	Water/Cu, Ag and Al	-- Φ = 0.5–3 vol.%	5000 W/m^2^	20–140 °C	180 L/h	- The nanofluids exhibited optimized perfor-mance at 2 vol.% concentration. The $\eta_{th}$ is augmented by 7.49, 7.08 and 4.97% for PV/T operated by water/Ag, water/Cu, and water/Al NFs, respectively, relative to water.

**Table 2:** Properties of the Material used for nanofluid Formulation.

Material	Parameter	Value
Therminol^®^55 (EASTMAN)	Appearance	Clear, yellow liquid
	Normal boiling point	351 (°C)
	Liquid density (20 °C)	872 (kg/m^3^)
	Thermal conductivity (20 °C)	0.1284 W/(m·K)
	Viscosity (20 °C)	41.6 (mPa·s)
Diamond (US Research Nanomaterials, Inc)	Color	Grey
	Purity	98.3%
	Size	3–10 (nm)
	Morphology	Spherical
	Density	0.16–0.18 (g/cm^3^)

**Table 3:** Specifications and properties of the PV/T system Nasrin, et al. [41].

Parameter	Values
Power	300 W
Dimensions	1955 mm × 982 mm × 36 mm
Weight of PV panel	20.5 kg
$h_{panel-tedlar}$	150 W/m^2^K
$h_{tedler-tubing}$	77 W/m^2^K
$h_{tubing-nanofluid}$	66 W/m^2^K
$A_{PV}$	0.9
$A_{tedlar}$	0.5
$Emissivity_{PV}$	0.99
$k_{EVA}$	0.311 W/m·K
$k_{PV}$	148 W/m·K
$k_{Tedlar}$	0.15 W/m·K

**Table 4:** Grid independency test.

S. No.	Mesh Size (Elements)	PV Temp. (°C)	Deviation (%)	Outlet Temp. (°C)	Deviation (%)
1	$2.5\times 10^{5}$	42.341	--	41.213	--
2	$4\times 10^{5}$	43.872	1.2	40.751	−1.13
3	$6\times 10^{5}$	44.003	0.29	40.254	−1.23
4	$8\times 10^{5}$	44.118	0.26	39.104	−2.94
5	$1.5\times 10^{6}$	45.200	2.3	38.889	−0.55
6	$3.5\times 10^{6}$	45.201	0.002	38.801	−0.22

**Table 5:** Zeta potential values of TH-55/DP nanofluids.

Concentration (wt.%)	Absolute Zeta Potential (mV)			
	At 25 °C	Uncertainty (%)	At 80 °C	Uncertainty (%)
0.001	45.13	<5	48.25	<5
0.05	41.21		44.98	
0.1	34.81		39.67	

**Table 6:** Validation of average cell Temperature.

Flowrate (LPM)	Cell Temperature (°C)		
	Present Research	Numerical Study Nasrin, et al. [41]	Experimental Study Rahman, et al. [8].
0.5	52.56	51.11	52.88
1	49.85	48.04	50.23
3	47.10	45.76	47.73

**Table 7:** Validation of Electrical and thermal efficiency.

Nanoparticle Concentration (Wt.%)	Electrical Efficiency (%)		Thermal Efficiency (%)	
	Present Research	Nasrin, et al. [61]	Present Research	Nasrin, et al. [61]
0.1%	11.38	11.96	63.8	73.5

## Data Availability

The data used for the analysis in this study are available within the article, while the datasets used or analyzed during the current study are available from the corresponding author upon reasonable request.

## Nomenclature

Nomenclature		*σ*	Stefan Boltzmann Constant, W/(m^2^·K^4^)
* A_c_*	area of collector (m^2^)	Subscripts	
* c_p_*	Specific heat (J/K)	*amb*	ambient
*FF*	field factor	*bf*	base fluid
*G*	solar radiation intensity (W/m^2^)	*el*	electrical
*H*	convective heat transfer coefficient (W/m^2^·K)	*in*	inlet
* I_sc_*	short circuit current (A)	*out*	outlet
*k_bf_*	thermal conductivity of base fluid (W/m·K)	*s*	solid particle
*k_nf_*	thermal conductivity of nanofluid (W/m·K)	*th*	thermal
*k_s_*	thermal conductivity of nanoparticle (W/m·K)	*nf*	Nanofluids
*P_el_*	electrical power output (W)	Abbreviations	
*P_th_*	thermal power output (W)	HPLC	high performance liquid chromatography
$Q_{conv}^{\prime }$	heat transfer due to convection (W)	NMR	nuclear magnetic resonance
$Q_{rad}^{\prime }$	heat loss due to radiation (W)	IC	ion chromatographic
*T*	temperature (K)	KF	Karl Fischer
*V_oc_*	open circuit voltage (V)	ND	Nano Diamond
Greeks		SEM	scanning electron microscopy
ζ	zeta potential, mV	FT-IR	Fourier-transform infrared spectroscopy
*Φ*	nanoparticle weight fraction	UV-Vis	ultraviolet–visible spectroscopy
*ρ*	density, kg/m^3^	TH-55	Therminol 55
*η*	efficiency	PV/T	photovoltaic thermal
*ε*	emissivity	UDF	user defined function
